# Scientific research and publications in health: aimed at the value of knowledge and solution-based approaches *solucionática*

**DOI:** 10.17843/rpmesp.2024.413.14277

**Published:** 2024-08-27

**Authors:** César Cabezas

**Affiliations:** 1 Instituto Nacional de Salud, Lima, Peru. Instituto Nacional de Salud Lima Peru

In the academic and scientific world, publications in indexed journals are considered one of the main indicators of research quality and relevance. Among these, journals classified in quartiles according to the “Journal Citation Reports” or “Scopus” ^1^^,^^2^ are seen as benchmarks of prestige and excellence. However, when reflecting on the fundamental purpose of science and the dissemination of knowledge, a crucial question arises: Is the prestige of the journal or the real contribution of the research more important?.

The prestige lies in the fact that journals ranked Q1 and Q2 occupy the highest positions in the impact rankings, which reflects their influence and recognition within the scientific community. Publishing in these journals increases the likelihood that the work will be reviewed by high-level experts and reach a global audience, increasing the visibility and potential influence of the researcher. The advantages of publishing in journals in these quartiles imply greater visibility and citation, recognition and validation of quality by the scientific community, as well as the possibility of establishing broader academic networks. However, these advantages come with significant challenges, such as high barriers to entry and review processes can be lengthy and competitive, often favoring studies with “positive” or higher impact results.

The quartile approach may have some limitations, including that excessive concentration in top quartile journals may limit the diversity of research that is actually required. Innovative studies or studies that do not follow the dominant trends may find it difficult to be accepted in these journals, such as publications on neglected infectious diseases or those of more regional interest. In addition, this approach may foster a culture of pressure to publish (publish or perish) that does not always prioritize the quality or relevance of the content. Thus, among the limitations we can mention the possible bias towards “fashionable” research, the exclusion of relevant studies with negative or null results, as well as the excessive pressure on young researchers who, in order to have better options, need to publish in these journals.

There are studies that show alternatives to the already known metrics for assessing the impact of health research, which address broad categories such as those related to primary research, influence on policy formulation, impact on health and health systems, impact on health and society, and broader economic impact. These studies have been developed in different contexts and countries, and although they share certain similarities, they also present significant variations in terms of their approaches, impact categories and metrics used, highlighting the importance of developing new metrics that more accurately capture the impact of research in different areas ^3^.

Since scientific publications are the result of research, we believe it is important to take into account the recommendations from the World Health Organization on health research, which include research on the problem, causes, solution, implementation and evaluation ^4^, which are carried out globally in the vast field of health. In this context, it is important to emphasize that scientific research has been and will continue to be the cornerstone on which knowledge is built; however, voices are heard and criticism arises that research advances do not translate quickly and efficiently into direct benefits for patients or the community in general, a condition that is not the result of a lack of effort or intentions, but of the intrinsically complex nature of the research and development process. The distance between a discovery in the laboratory and its application in the field can be long and fraught with technical, financial and regulatory obstacles. Of course, it should be mentioned that we cannot expect an immediate impact from basic science research, which is important and continues its process, remembering Pasteur, who stated that “there is no applied science, but applications of science”.

In this regard, we must recall the message from Peruvian epidemiologist Joaquín Cornejo Ubilluz, who as a response to the prevalent problematic situation, coined a term in Spanish: “*solucionática*” (the term will be referred to as “solutionatics” in English, meaning solution-based approach), which is used in the medical field; and even though it does not appear in the Dictionary of the RAE (Royal Spanish Academy), it is easy to understand because it accurately encompasses the need for knowledge as a result of research not only to remain in laboratories, in the pages of scientific journals, or in very attractive prototypes, but to be translated into practical and tangible solutions for society. This is what Cornejo-Ubilluz intuitively proposed as solutionatic, when he shared discussions in the classrooms of San Marcos and in the field. We believe this concept should imply a proactive and results-oriented approach, where each phase of research is planned with practical application in mind, with solutionatics not being simply the translation of theory into practice, but the optimization of the entire research process so that each discovery, each innovation, has a clear and defined path to its effective implementation.

A clear example of the path to solutionatics is translational research ^5^, increasingly addressed as a field that precisely focuses on “translating” findings from basic research into practical solutions, such as treatments, therapies, and public health policies. Thus, solutionatics is a concept that should be considered when conducting health research in order to guide it towards real and positive impacts on society.

In this context, instead of the ranking of the journal, the true value of a research lies in its contribution to knowledge and its potential to generate positive changes. Science should be a collaborative effort that seeks the common good and not just individual recognition. Publishing in lower impact journals, as long as they maintain scientific rigor, can be valuable, particularly when the research is relevant to local contexts or in languages other than English.

**Figure 1:**
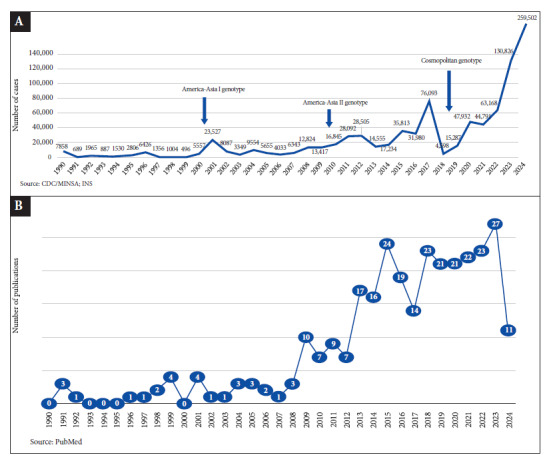
Dengue cases (A). Publications on dengue in Q1, Q2, and others (B) during the period from 1990 to 2024.

In this context, we should strive to ensure that the scientific knowledge contained in publications is available in both Spanish and English, broadening access and promoting greater inclusiveness. On the other hand, it is worth mentioning the contribution of Dr. Tu You You, who at first published internally, even in Chinese, which was her native language. However, her discovery of the therapeutic effect of artemisinin derived from *Artemisia annua* L. (Qinghao) for malaria was so important that she was awarded the Nobel Prize in Medicine in 2015 ^6^. Additionally, contrary to what has been mentioned, the number of publications on dengue in Peru in high impact journals has shown a sustained increase, parallel to dengue epidemics since the 1990s (Figure 1), evidencing a gap between research and actually solving the studied problem, which should be discussed among all actors in this phenomenon to seek solutionatics, considering that the common goal is to prevent and control this serious health problem.

Ultimately, it is good to publish in journals classified in the top quartiles. However, the impact of a publication should not be measured solely by the prestige of the journal in which it appears but by the real contribution it offers to its field of study and society. It is essential to value diverse approaches and the accessibility of knowledge, promoting more inclusive and representative science. High-impact journals will continue to be a pillar in scientific communication, but they should not be the only way to validate the quality of research. We encourage researchers to consider both the prestige and the purpose and impact of their work, contributing to a more equitable and enriching scientific environment to improve individual and collective health within a framework of local, regional, and global health.
