# Supplementary material for: Scientific research and publications in health: aimed at the value of knowledge and solution-based approaches solucionática
Source: Rev Peru Med Exp Salud Publica. 2024 Aug 27;41(3):236–8. doi: 10.17843/rpmesp.2024.413.14277 (PMC11495921; doi:10.17843/rpmesp.2024.413.14277)
Supplement: Translation

# Translation: Investigaciones y publicaciones científicas en salud: un enfoque en el valor del conocimiento y la solucionática

**Authors:** César Cabezas

**Affiliations:** 1 Instituto Nacional de Salud, Lima, Peru.

En el mundo académico y científico, las publicaciones en revistas indexadas se consideran uno de los principales indicadores de calidad y relevancia de la investigación. Entre estas, las revistas clasificadas en cuartiles según el «Journal Citation Reports» o «Scopus» ^1^^,^^2^ son vistas como referentes de prestigio y excelencia. Sin embargo, al reflexionar sobre el propósito fundamental de la ciencia y la difusión del conocimiento, surge una pregunta crucial ¿Es más importante el prestigio de la revista o el aporte real de la investigación?.

El prestigio radica en que las revistas catalogadas en Q1 y Q2 ocupan las posiciones más altas en los ránquines de impacto, lo que refleja su influencia y reconocimiento dentro de la comunidad científica. Publicar en estas revistas aumenta la probabilidad de que el trabajo sea revisado por expertos de alto nivel y que alcance una audiencia global, aumentando la visibilidad y potencial influencia del investigador. Las ventajas de publicar en revistas en estos cuartiles implican una mayor visibilidad y citación, reconocimiento y validación de la calidad por parte de la comunidad científica, así como la posibilidad de establecer redes académicas más amplias. Sin embargo, estas ventajas vienen acompañadas de desafíos significativos, como que las barreras de entrada son altas y los procesos de revisión pueden ser largos y competitivos, a menudo favoreciendo estudios con resultados «positivos» o de mayor impacto.

El enfoque de cuartiles podría tener algunas limitaciones, entre ellas podemos mencionar, que la excesiva concentración en las revistas de los primeros cuartiles puede limitar la diversidad de la investigación que realmente se requiere. Estudios innovadores o que no siguen las tendencias dominantes pueden encontrar dificultades para ser aceptados en estas publicaciones, como por ejemplo publicaciones sobre enfermedades infecciosas desatendidas o de interés más regional. Además, este enfoque puede fomentar una cultura de presión por publicar (publish or perish) que no siempre prioriza la calidad o relevancia del contenido. De este modo, entre las limitaciones podemos mencionar el posible sesgo hacia investigaciones «de moda», la exclusión de estudios relevantes con resultados negativos o nulos, así como la presión excesiva sobre los investigadores jóvenes que, para tener mejores opciones, requieren haber publicado en estas revistas.

Hay estudios que muestran alternativas a las métricas ya conocidas para evaluar el impacto de la investigación en salud, que abordan amplias categorías como las relacionadas con la investigación primaria, influencia en la formulación de políticas, impacto en la salud y los sistemas de salud, impacto en la salud y la sociedad, e impacto económico más amplio. Estos estudios se han desarrollado en diversos contextos y países, y aunque comparten ciertas similitudes, también presentan variaciones significativas en cuanto a sus enfoques, categorías de impacto y métricas utilizadas, debiendo destacar la importancia de desarrollar nuevas métricas que capturen de manera más precisa el impacto de la investigación en diversas áreas ^3^.

Siendo las publicaciones científicas un producto resultante de las investigaciones, creemos importante tener en cuenta las recomendaciones de la Organización Mundial de la Salud sobre las investigaciones en salud que incluyen las referidas al problema, a las causas, a la solución, la implementación y la evaluación ^4^ que de hecho globalmente se realizan en el vasto campo de la salud. En ese contexto importa enfatizar que la investigación científica ha sido y seguirá siendo la piedra angular sobre la cual se construye el conocimiento; sin embargo, se oyen voces y surge la crítica de que los avances de las investigaciones no se traducen de manera rápida y eficiente en beneficios directos para los pacientes o la comunidad en general, condición que no es fruto de la falta de esfuerzos o intenciones, sino de la naturaleza intrínsecamente compleja del proceso de investigación y desarrollo. La distancia entre un descubrimiento en el laboratorio y su aplicación en el campo puede ser larga y llena de obstáculos, tanto técnicos, financieros, como regulatorios. Evidentemente, es preciso mencionar que no podemos esperar un impacto inmediato de las investigaciones en ciencia básica que son importantes y siguen su proceso, recordando también a Pasteur, quien afirmaba que «no existe ciencia aplicada, sino aplicaciones de la ciencia».

En esa línea debemos recordar el mensaje del epidemiólogo peruano Joaquín Cornejo Ubilluz, quien acuñó como respuesta a la muy prevalente situación problemática, la solucionática, término que se usa en el ámbito médico y aun cuando no aparece en el Diccionario de la RAE, no es difícil entenderlo por que engloba de manera precisa la necesidad de que el conocimiento generado producto de la investigación no solo se quede en los laboratorios, en las páginas de revistas científicas, o en prototipos muy atractivos, sino que se traduzca en soluciones prácticas y tangibles para la sociedad. Es lo que intuitivamente proponía el maestro Cornejo-Ubilluz como la solucionática, cuando compartía discusiones en las aulas de San Marcos y en el campo. Este concepto, creemos que debe implicar un enfoque proactivo y orientado a resultados, donde cada fase de la investigación se planifica con la aplicación práctica en mente, no siendo la solucionática simplemente la traducción de la teoría en práctica, sino la optimización de todo el proceso de investigación para que cada descubrimiento, cada innovación, tenga un camino claro y definido hacia su implementación efectiva.

Un claro ejemplo de la ruta a la solucionática, es la investigación traslacional ^5^, últimamente más abordada, como un campo que se dedica precisamente a «traducir» los hallazgos de la investigación básica en soluciones prácticas, como tratamientos, terapias y políticas de salud pública, de modo que la solucionática es un concepto que debiera tenerse en cuenta cuando se efectúa investigación en salud para orientar a esta última en sus objetivos hacia un impacto real y positivo en la sociedad.

En ese contexto, más allá del ranquin de la revista, el verdadero valor de una investigación radica en su contribución al conocimiento y su potencial para generar cambios positivos. La ciencia debe ser un esfuerzo colaborativo que busque el bienestar común y no solo el reconocimiento individual. Publicar en revistas clasificadas de menor impacto, siempre que mantengan rigurosidad científica, puede ser valioso, especialmente cuando se trata de investigaciones relevantes para contextos locales o en idiomas distintos al inglés.

En nuestro medio se debe procurar y asegurar que el conocimiento científico contenido en las publicaciones esté disponible tanto en idioma español como en inglés, ampliando el acceso y fomentando una mayor inclusividad. Por otro lado, no está demás comentar el aporte de la Dra. Tu You You, quien inicialmente tuvo una publicación interna incluso en chino, que era su lengua materna, pero su hallazgo sobre el efecto terapéutico de la artemisinina derivada de la *Artemisia annua L*. (Qinghao) para la malaria fue tan importante que en 2015 fue galardonada con el Premio Nobel de Medicina ^6^. También comentar que contrariamente a lo mencionado, el número de publicaciones sobre dengue en el Perú en revistas de alto impacto tienen un ascenso sostenido, paralelo a las epidemias de dengue desde la década de los 90 (Figura 1), evidenciando una fractura entre la investigación y la solución del problema estudiado, que debiera discutirse entre todos los actores de este fenómeno para buscar la solucionática, considerando que el objetivo común es el de prevenir y controlar este serio problema de salud.

**Figura 1:** Casos de dengue (A) . Publicaciones sobre dengue en Q1, Q2 y demás (B) en el período 1990 a 2024.

En última instancia, es bueno publicar en revistas clasificadas en los primeros cuartiles. Sin embargo, el impacto de una publicación no debería medirse únicamente por el prestigio de la revista en la que aparece, sino por el aporte real que ofrece a su campo de estudio y a la sociedad. Es esencial valorar la diversidad de enfoques y la accesibilidad del conocimiento, promoviendo una ciencia más inclusiva y representativa. Las revistas clasificadas de alto impacto seguirán siendo un pilar en la comunicación científica, pero no deben ser la única vía para validar la calidad de la investigación. Alentamos a los investigadores que puedan considerar tanto el prestigio como el propósito y el impacto de su trabajo, contribuyendo a un ambiente científico más equitativo y enriquecedor, para contribuir a mejorar la salud individual y colectiva en un marco de salud local, regional y global.
